# CT evaluation of pediatric craniocervical distraction injuries: a comprehensive review of morphometric parameters and practical applications

**DOI:** 10.1007/s00247-026-06583-5

**Published:** 2026-04-07

**Authors:** Rithika Sriram, Yashasvi Shukla, Laura L. Hayes, Vinay V. R. Kandula

**Affiliations:** 1 Department of Imaging, Nemours Children’s Hospital, 1600 Rockland Rd, Wilmington, DE 19803 USA; 2 Children’s Healthcare of Atlanta, Atlanta, United States

**Keywords:** Atlanto-occipital  distraction, Pediatric trauma, Occipital condyle-C1 interval, Atlanto-dental interval, Computed tomography

## Abstract

Craniocervical distraction injuries in children are uncommon but can be fatal, often resulting from high-impact trauma. Because these injuries predominantly affect ligaments, CT findings may be subtle or may occur without obvious fracture or malalignment. This review consolidates the relevant anatomy and key CT-based morphometric parameters essential for recognizing such injuries, including the basion–dens interval, Occipital condyle–C1 interval, atlanto-dental interval, and interspinous ratio. The article integrates normative data and updated measurement thresholds from recent literature, emphasizing their age-related variability and diagnostic implications. The purpose of this review is to provide pediatric radiologists with a consolidated resource that integrates the latest CT measurements and interpretive guidelines for quick and reliable consultation. By improving awareness of subtle quantitative deviations and anatomical nuances, this review aims to enhance diagnostic accuracy and promote early recognition of potentially unstable craniocervical injuries in pediatric trauma assessment.

## Introduction

Craniocervical junction (CCJ) distraction injuries in children, though uncommon in the general population, are clinically significant injuries that represent a notable subset of pediatric cervical spine trauma. These injuries are predominantly ligamentous, often occurring without fractures. Consequently, findings on computed tomography (CT) can be subtle, posing a significant diagnostic challenge for radiologists. Experience from high-volume trauma centers indicates that these dissociation injuries are frequently overlooked. Notably, a recent 2024 review highlighted that diagnosis is delayed beyond 24 hours in up to 25% of cases [1]. Despite the critical importance of early detection, literature lacks a comprehensive, age-specific reference compiling all CT-based morphometric parameters and their normal values for children. Existing resources are fragmented, and radiologists face difficulties in applying measurements consistently across varying ages and developmental stages.

This review aims to address this gap by:

1.  Summarizing the key CT parameters for assessing pediatric craniocervical distraction injury
2.  Providing updated, age-specific cut-off values derived from recent literature
3.  Illustrating the practical application of these measurements with representative cases
4.  Highlighting common challenges in measurement and suggesting strategies to improve diagnostic accuracy and consistency

By consolidating these measurements and interpretive guidelines into a single, clinically accessible reference, we seek to enhance diagnostic accuracy, facilitate timely intervention, and ultimately improve outcomes for children with suspected craniocervical distraction injuries.

It is important to note that normal values for these parameters can vary significantly throughout childhood. Baker et al. [2] highlighted that caution is necessary when applying rigid measurement thresholds across different pediatric age groups due to developmental changes. Furthermore, measurement variability is a known challenge. The occipital condyle-C1 interval (CCI), in particular, can exhibit variability due to difficulties in consistent landmark identification and age-related changes*.* The metrics included in this review were selected based on their established prevalence in the literature and diagnostic utility. We have specifically updated the review to reflect the latest measurement methods currently in clinical practice.

## Anatomy of the craniocervical junction

A thorough understanding of the pediatric craniocervical junction (CCJ) anatomy and biomechanics is fundamental for accurate assessment of trauma imaging. The CCJ, comprising the occipital bone, atlas (C1), and axis (C2), with their associated ligaments, forms a specialized osteoligamentous unit that provides neck stability and mobility while safeguarding critical neurovascular structures, including the brainstem, spinal cord, and vertebral arteries [3]. The pediatric CCJ is particularly susceptible to distraction injuries due to unique anatomical features, including inherent ligamentous laxity, incomplete ossification, and relatively shallow, horizontally oriented facet joints.

**Fig. 1:**
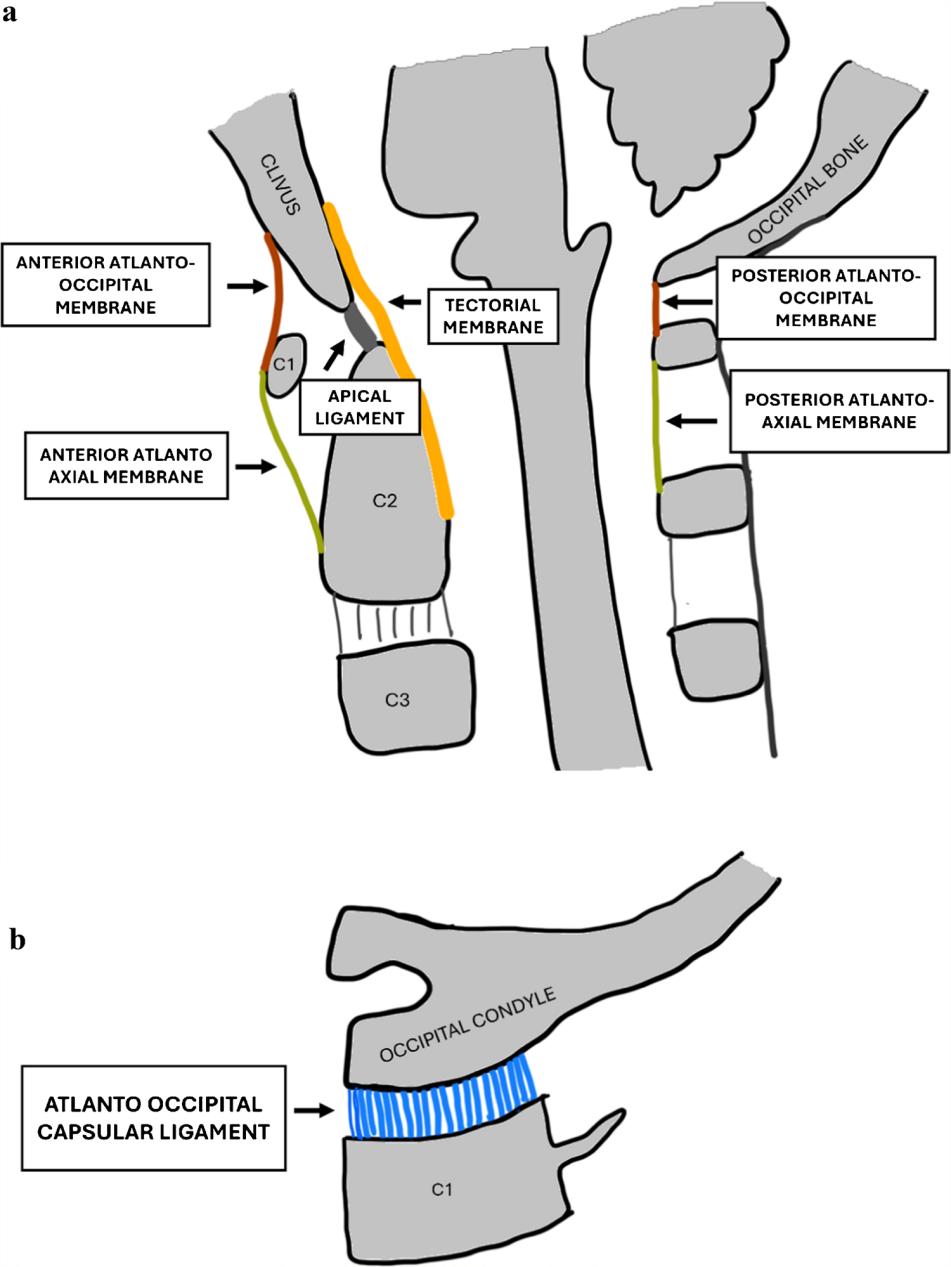
**(a**)Sagittal schematic diagram of the craniocervical junction. (**b)** Parasagittal schematic diagram showing the atlanto-occipital capsular ligaments between the occipital condyle and C1

**Fig. 2:**
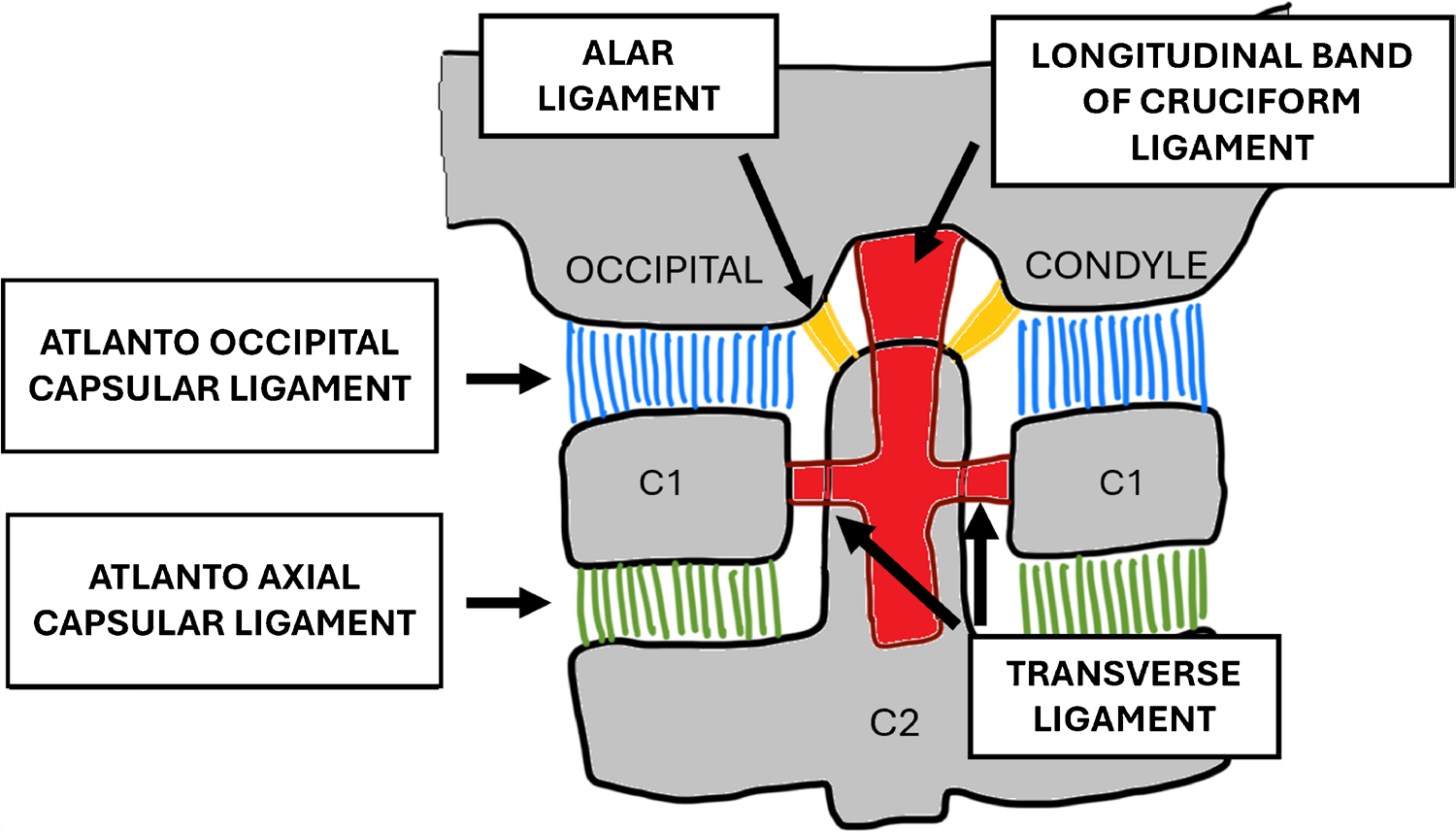
Coronal schematic diagram of the craniocervical junction with the major ligaments

To facilitate anatomical comprehension and its application in CT interpretation, schematic diagrams in Figs. 1a and b and 2 illustrate the key ligaments in sagittal and coronal planes. These multi-planar views help radiologists correlate ligament anatomy with imaging findings. Each ligament is briefly described to provide a practical reference for measurement and diagnosis.

The CCJ consists of the atlanto-occipital joints and the atlanto-axial joints.

The atlanto-occipital and atlanto-axial joints are stabilized primarily by five ligamentous structures: the occipital–atlanto capsular ligaments, atlanto-axial capsular ligaments, transverse ligament, tectorial membrane, and alar ligaments.

### Capsular ligaments

The capsular ligaments of the atlanto-occipital (OC-C1) and atlanto-axial (C1-C2) joints are now recognized as the primary stabilizers of the craniocervical junction against distraction [4]. Atlanto-occipital (AO) capsules attach the occipital condyles to the superior articular facets of the atlas (C1), and the atlanto-axial (AA) capsules secure the inferior articular facets of C1 to the superior facets of the axis (C2). Recent finite element analyses by Phuntsok et al. [4] have demonstrated that the OC-C1 capsular ligaments are the principal restraint against vertical distraction at the atlanto-occipital joint, while the C1-C2 capsular ligaments work in concert with the transverse ligament to stabilize the atlanto-axial joint. Injury to these structures is a hallmark of instability.

### Cruciform ligament

The cruciform or cruciate ligament serves as the principal stabilizer of the atlanto-axial joint. Structurally, it is cross-shaped and situated posterior to the dens, comprising superior and inferior longitudinal bands as well as a bilateral transverse band also known as the transverse ligament [5]. The vertical (longitudinal) band of the ligament attaches from the posterior aspect of the dens to the clivus, positioned between the apical ligament anteriorly and the tectorial membrane posteriorly. These fibers are not distinguishable as separate entities on CT or MRI. The transverse ligament is the strongest and thickest portion of the ligament, appearing as a hyperdense band posterior to the dens on axial CT images and as a T2 hypointense band on MRI [6]. A widening of the atlanto-dental interval is a key radiologic indicator of transverse ligament injury [7].

### Tectorial membrane

The tectorial membrane is a slender ligament, approximately 1 mm in thickness, that extends from the clivus to the posterior aspect of the dens, forming a sling-like structure. Inferiorly, at the C2–C3 intervertebral level, it continues as the posterior longitudinal ligament. This membrane is closely adherent to the dura mater posterior to the clivus [8]. On computed tomography (CT), the membrane is typically too thin to be visualized distinctly from the dura. As a result, the dura mater, tectorial membrane, and cruciform ligament are often observed as a single complex. The presence of retroclival hyperdensity on imaging may be indicative of injury. Retroclival hyperdensity may represent epidural hematoma (typically more significant regarding ligamentous stripping) or subdural hematoma. MRI is valuable for distinguishing the exact compartment and relationship to the tectorial membrane [9]. While isolated injury to the tectorial membrane may not always result in frank mechanical instability, it is a marker of significant distraction force and is frequently associated with injury to the cruciform or alar ligaments.

### Alar ligaments

The alar ligaments are paired structures that connect the superior half of the lateral dens to regions just medial to the occipital condyles, coursing in a nearly horizontal orientation [10]. This anatomical alignment enables the alar ligaments to restrict axial and lateral rotation, as well as flexion in the sagittal plane. Isolated unilateral alar ligament injury has been documented in pediatric patients, typically resulting from abrupt contralateral rotation or hyperflexion of the neck at force levels insufficient to damage bone, the transverse ligament, or the tectorial membrane [11]. Traumatic avulsion fractures at the alar ligament insertion on the occipital condyle are classified as type 3 Anderson and Montesano fractures and are associated with a high incidence of craniocervical instability [12].

## Ancillary ligamentous structures

### Anterior atlanto-occipital and atlanto-axial membrane

The anterior atlanto-occipital and atlanto-axial membranes are fibroelastic structures that originate at the anterior margin of the clivus and extend to the anterior arch of the atlas (C1) and from the anterior arch of C1 to the anterior surface of C2 respectively. Inferiorly, it continues as the anterior longitudinal ligament, which courses along the anterior aspect of the vertebral bodies [5].

### Apical ligament

The apical ligament extends from the basion (anterior margin of the foramen magnum) to the tip of the odontoid process. On CT, the distinct fibrous bands of the apical ligament are rarely visualized directly. Importantly, the apical ligament is not considered essential for craniocervical junction stability. Anatomical studies in adult cadavers have identified congenital absence (aplasia) of this ligament in approximately 20% of individuals [13].

### Posterior atlanto-occipital and atlanto-axial membrane complex 

The posterior atlanto-occipital and atlanto-axial membrane complex (PAOMc) is comprised of two distinct components: (1) the posterior atlanto-occipital membrane, which spans the interval between the occipital bone and the atlas (C1), and (2) the posterior atlanto-axial membrane, which extends from the atlas (C1) to the axis (C2). Anatomically, the anterior boundary of the PAOMc attaches directly to the posterior spinal dura at the level of C1, while its posterior boundary forms a myoligamentous complex with the tendons of the rectus capitis posterior major and minor muscles, as well as fibers of the ligamentum nuchae.

## What are distraction injuries and why are children more affected?

Craniocervical distraction is defined as a dislocation or subluxation of the occipital bone from the cervical spine, or between the upper cervical vertebrae, resulting from disruption of the bony and/or ligamentous stabilizers of the craniocervical junction (CCJ). This injury is life-threatening and occurs as a consequence of significant hyperflexion–hyperextension forces applied to the CCJ [14]. High-speed motor vehicle collisions and pedestrian vehicle accidents represent the most common mechanisms of injury. Craniocervical distraction frequently results from a combination of forces including hyperextension, hyperflexion, and lateral flexion.

Among trauma victims, children exhibit a significantly higher predilection for craniocervical distraction compared to adults, owing to the unique anatomical and biomechanical characteristics of the pediatric CCJ. Pediatric patients have a proportionally larger head, which shifts the fulcrum of cervical motion cephalad, and have underdeveloped cervical musculature leading to a lower biomechanical threshold for distraction injuries. Additional factors contributing to increased vulnerability include ligamentous laxity, shallow and flat joint surfaces, incomplete ossification, and a higher fulcrum of motion (C2–C3 in children versus C5–C6 in adults). Collectively, these features reduce intrinsic stability, rendering the pediatric CCJ particularly susceptible to high-energy distraction injuries [15].

As children age, the craniocervical junction ossifies and musculature strengthens; therefore, injury patterns in later childhood begin to demonstrate a more adult pattern where fractures are more common than pure ligamentous injuries.

## Clinical presentation of pediatric craniocervical distraction injuries

Pediatric craniocervical distraction injuries frequently present with severe clinical manifestations, including apnea, respiratory distress, quadriparesis or quadriplegia, and hemodynamic instability [16]. Distraction injuries range from subtle subluxations to complete dislocations, with the latter typically resulting in high mortality*.* Associated findings often include cranial nerve deficits and traumatic brain injury [17].

Less severe cases may present with persistent neck pain, torticollis, or subtle neurological deficits, which can delay diagnosis. Survivors of these injuries generally exhibit lower Injury Severity Scores and higher Glasgow Coma Scale scores [18].

Early detection is essential to prevent neurological deterioration. In pediatric trauma cases, computed tomography (CT) should be carefully evaluated for subtle distraction injuries, and any suspicious findings should prompt magnetic resonance imaging (MRI) for ligamentous assessment. Given the subtle nature of these injuries, imaging remains central to the diagnosis. This review compiles essential CT measurements and age-specific thresholds to facilitate recognition of pediatric craniocervical distraction injuries.

## Imaging technique and diagnostic pathway

The assessment of pediatric spine trauma has been standardized through the American College of Radiology (ACR) Appropriateness Criteria [19] which stratifies imaging based on patient age, mechanism of injury, and clinical findings. A critical distinction in these guidelines is the management of children younger than 3 years of age. In this cohort, the ACR generally considers CT of the cervical spine to be usually not appropriate as an initial screening tool. This recommendation stems from the increased radiation sensitivity of the thyroid gland and bone marrow in toddlers, as well as the high prevalence of purely ligamentous injuries which are poorly characterized by CT. For these young children, if advanced imaging is clinically indicated due to high-risk mechanisms or neurological concerns, MRI is the preferred modality.

For children aged 3 years and older who meet high-risk criteria—such as those involved in high-speed motor vehicle accidents, patients with a Glasgow Coma Scale score of 12 or less, or those with focal neurological deficits—high-resolution CT is the indicated initial imaging modality. While CT is highly sensitive for osseous fractures, its sensitivity for distraction injuries remains limited. Therefore, adhering to rigorous technical parameters and systematic review is essential to avoid missing subtle ligamentous disruptions.

## Imaging protocols and techniques

### Computed tomography (CT)

High-resolution volumetric acquisition (≤0.625 mm) is the standard for pediatric cervical spine evaluation. Multi-planar reformats (MPR) in sagittal and coronal planes (typically 2-mm thickness) are mandatory, as axial images alone are insufficient for assessing distraction injuries. Crucially, images must be reviewed in all three planes.

Given the high association between head trauma and upper cervical injury, the field of view for pediatric head CTs should ideally extend caudally to include the C3 level. Conversely, cervical spine CT reconstructions must extend cranially to include the entire clivus and foramen magnum to ensure the CCJ is fully evaluable.

### Practical pearls

1. Along with the specific morphometric measurements detailed below, qualitative assessment of the soft tissues is critical. Standard bone windows can obscure subtle pathology; therefore, reviewing images with widened window widths (e.g., *W* 4,000/*L* 400) or dedicated soft-tissue kernels is essential for detecting subtle retroclival or interspinous hematomas, which often serve as the earliest indirect signs of ligamentous injury [20].
2. Craniometric measurements may appear normal even in severe injury if the distraction has spontaneously reduced before imaging. In such cases, static CT metrics may be deceptive, and diagnosis relies on identifying secondary signs of injury.
3. Ill-fitting cervical collars can induce artificial subluxation or distraction, particularly in small children. Radiologists should be wary of abnormal measurements that do not correlate with soft-tissue injury, as these may represent a positioning artifact rather than true pathology.

### Magnetic resonance imaging (MRI)

MRI is indicated when CT findings are equivocal, when there is a discrepancy between clinical status and CT findings such as in spinal cord injury without radiographic abnormality (SCIWORA), or when morphometric values (such as the CCI, ADI, or BDI) exceed age-appropriate thresholds [19]. Furthermore, a lower threshold for obtaining an MRI should be considered in very young children due to the inherent difficulties in obtaining accurate CT measurements, less robust normative data available for this specific age group, and the challenges associated with their clinical assessment. A rapid trauma MRI protocol including fat-saturated sequences is recommended to minimize sedation time while maximizing diagnostic yield.

## CT measurements in the sagittal plane

The systematic evaluation of the craniocervical junction in the sagittal plane has shifted from traditional distance-based lines toward joint-based metrics that directly interrogate the primary stabilizers of the spine. The most critical parameter to assess in the sagittal plane is the occipital condyle–C1 interval, followed by the interspinous distance ratio, anterior atlanto-dental interval, and ancillary measurements like the basion-dens or basion-cartilaginous dens interval and Powers ratio.

### 1. Occipital condyle–C1 interval

The occipital condyle–C1 interval (CCI) is a \gittal and coronal CT images to assess the integrity of the atlanto-occipital joint. In one of the largest pediatric cohorts evaluated by CT (*n*=597), Ravindra et al. [21] established thresholds for CCI that optimize diagnostic accuracy for atlanto-occipital dissociation (AOD). Using combined results for both measurements, they demonstrated that a sagittal CCI ≥2.5 mm or a coronal CCI ≥3.5 mm predicted AOD with 95% sensitivity, 73% specificity, 10.3% positive predictive value, and 99% negative predictive value. Although the authors found a correlation between normal measurements and age, establishing age-specific thresholds for children under 5 remained outside the scope of their study.

**Fig. 3:**
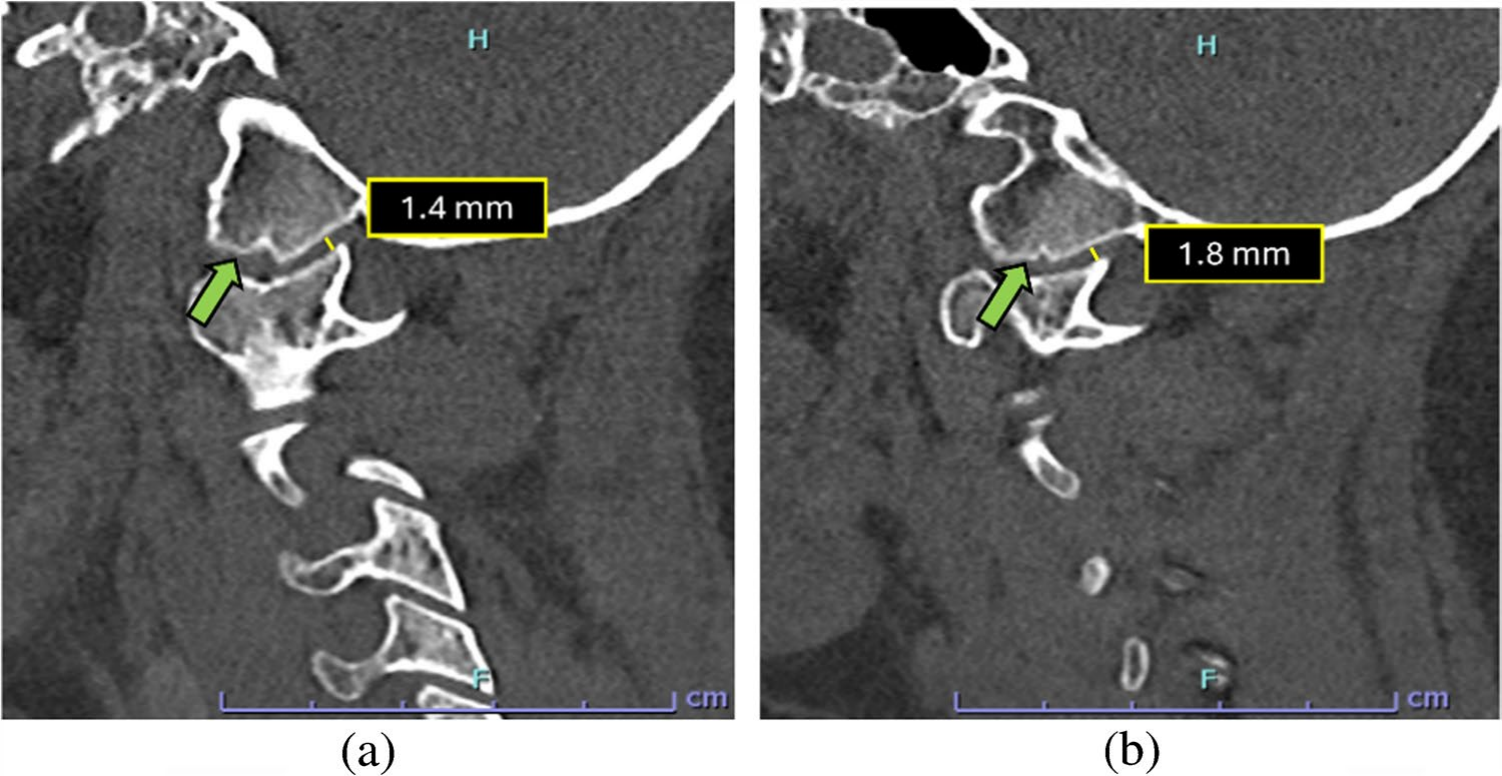
Parasagittal CT images of the cervical spine: right (**a**) and left (**b**) demonstrating normal occipital condyle–C1 interval (CCI) measurements. The minimum joint space at the lateral-most aspect of the joint in the parasagittal section (normal <2.5 mm) is measured, with care taken to exclude the medial occipital notch (*green arrow*) to avoid false-positive interpretation. The medial occipital notch is shown on either side by the *green arrow*. The morphology of the occipital notch is different on either side and this is a common finding

To ensure diagnostic accuracy, the joint distance should be measured at the lateral-most aspect, representing the shortest distance of the articulation. This specific technique—typically performed along the anterior or posterior aspect of the joint in the sagittal plane—is required to exclude the medial occipital notch [22]. Although this developmental variant is most useful for guiding coronal assessment, it is prevalent in children aged 1–12 years and can cause measurement asymmetry if one side of the joint is inadvertently sampled within it (Fig. 3). Therefore, focusing on the lateral aspect ensures a consistent and reliable metric for stability [23].

It is important to note that the sagittal plane should be primarily reserved for qualitative assessment. Due to the sloping anatomy of the condyles and high variability in slice selection, quantitative measurements in this plane are often inconsistent. Instead, radiologists should evaluate sagittal images for joint congruity, looking for anterior-posterior asymmetry or widening as clinical indicators of instability. Conversely, the coronal plane serves as the quantitative gold standard, allowing for precise measurement of the lateral joint space, which remains relatively constant across age groups [23].

**Fig. 4:**
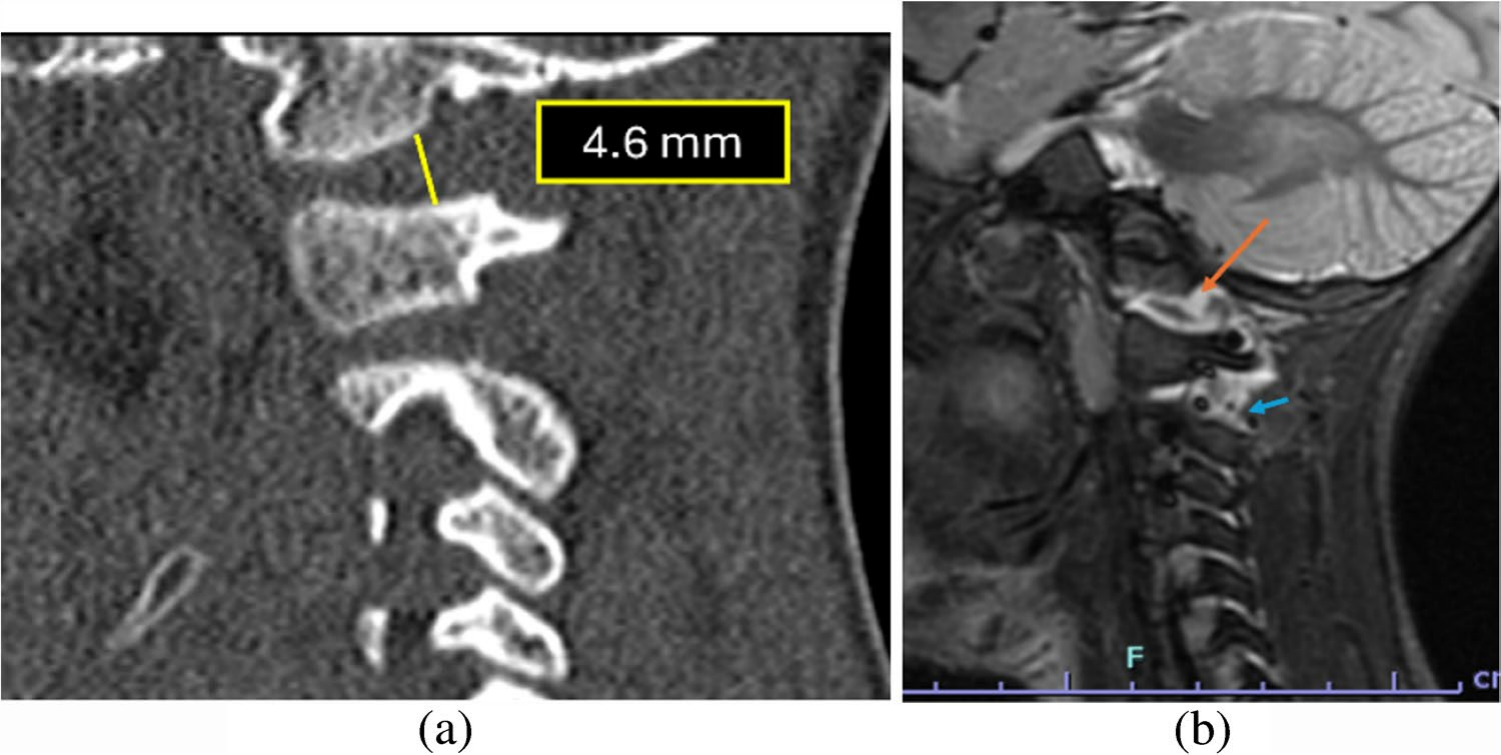
A 4-year-old child struck by a truck. (**a**) Sagittal CT of the cervical spine demonstrates an abnormally increased occipital condyle–C1 interval (CCI) on the right [N<2.5 mm] with a V-shaped widening of the joint suggestive of atlanto-occipital dissociation. (**b**) Subsequent MRI corroborates a distraction-type injury, showing fluid within the widened condyle–C1 joints (*orange arrow*) and adjacent soft-tissue edema, consistent with ligamentous disruption at the craniocervical junction. There is also fluid seen in the interspinous region at C1-C2 interval (*blue arrow*) indicative of soft-tissue injury

Figure 4 depicts a case with an increased occipital condyle-C1 interval along with its corresponding MRI correlation.

### 2. Interspinous distance ratio

**Fig. 5:**
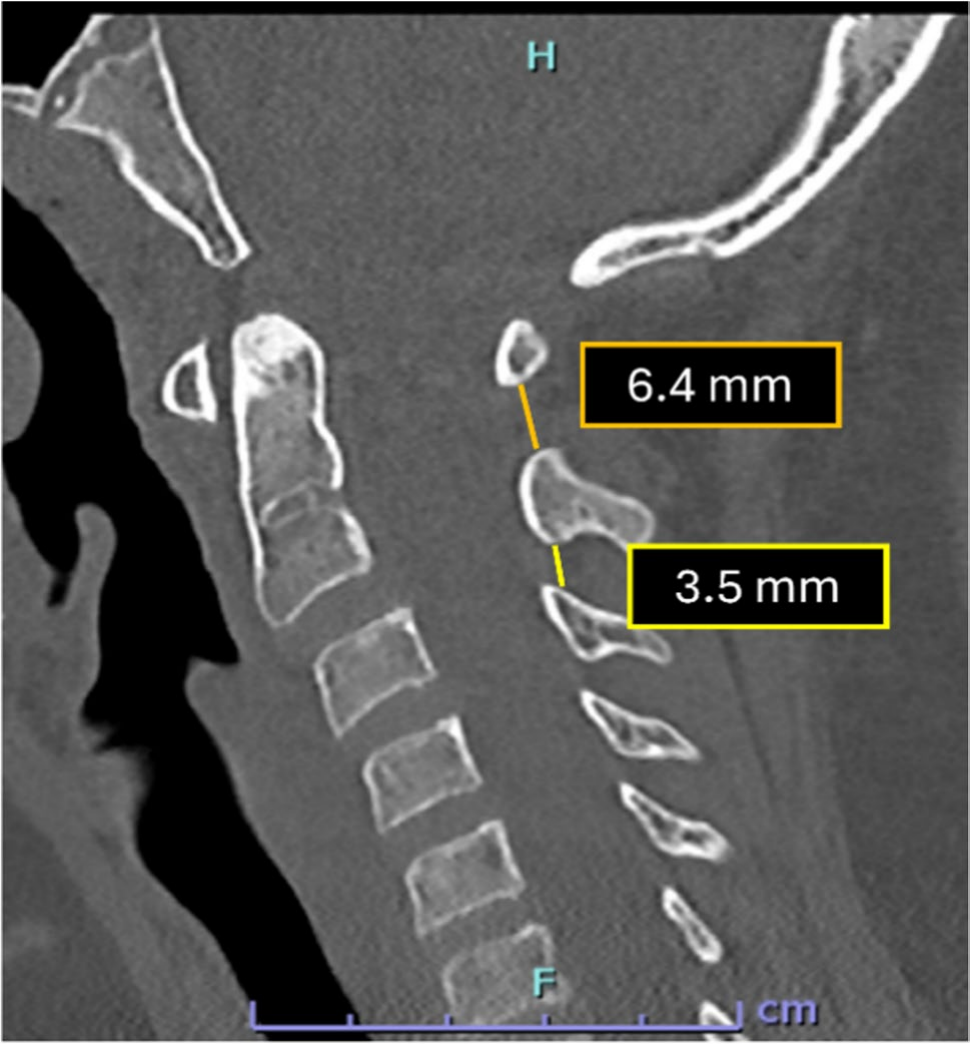
Sagittal CT of the cervical spine in a 7-year-old child demonstrating interspinous distance measurements at the C1–2 and C2–3 levels. Here, the measurement is obtained by dividing 6.4 mm (C1-C2 distance) by 3.5 mm (C2-C3) measuring 1.8 (normal ratio <2.5). Measurements are obtained in the midline, extending from the posterior cortex of the inferior spinous process to the superior tip of the adjacent superior spinous process, following reference thresholds originally established on lateral radiographs

**Fig. 6:**
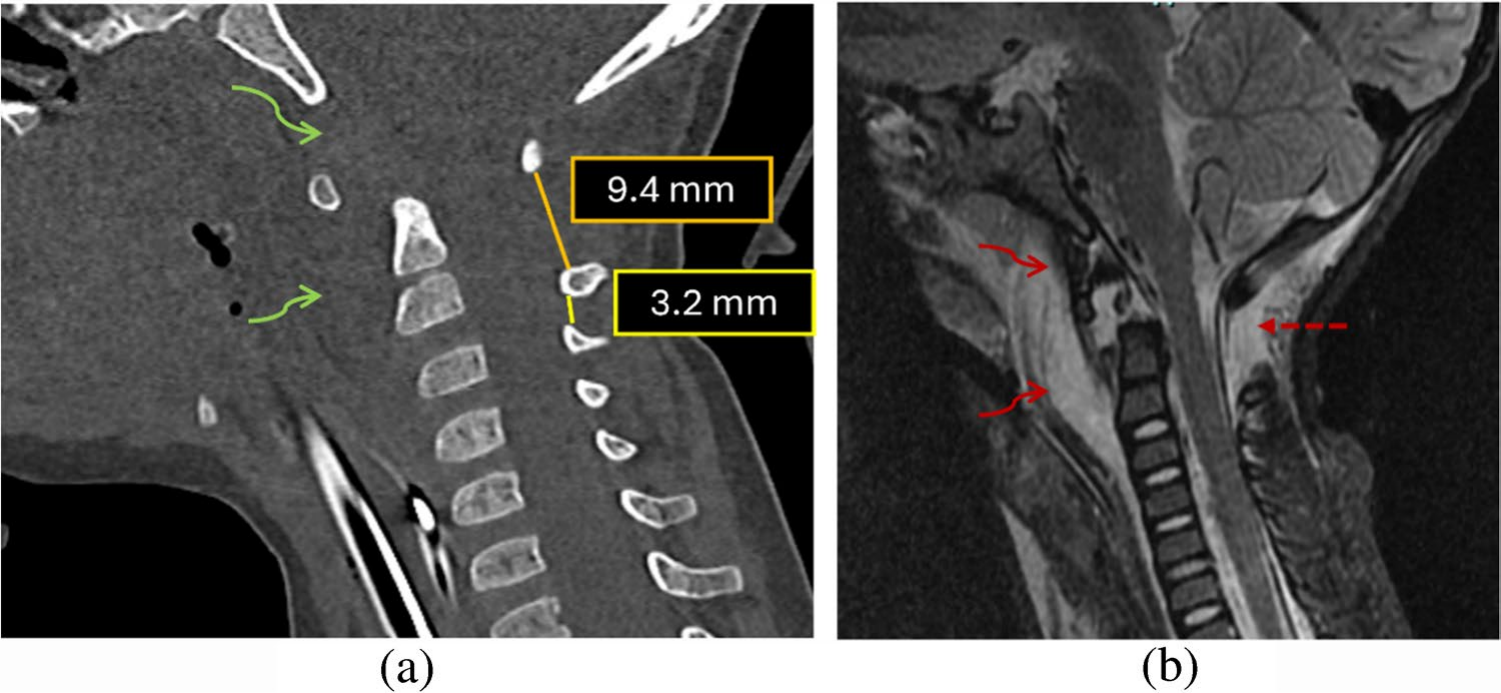
3-year-old child with a history of trauma. (**a)** On sagittal CT of the cervical spine, increased interspinous distance between C1-C2 and C2-C3 was noted, measuring 2.9  (ratio 9.4/3.2), along with increased prevertebral soft-tissue (*curved green arrow*) and widened anterior atlanto-dental interval raising concern for a distraction injury. (**b)** Subsequent MRI of the cervical spine demonstrated fracture of the cartilaginous dens with edema in the basion-dens interval and in the prevertebral space (*curved red arrows*). There was fluid in the interspinous region between C1 and C2 with widening of this interval (*dashed red arrows*), also concerning for distraction injury. The child was re-immobilized in a cervical collar and exhibited gradual clinical recovery

The interspinous distance ratio is a radiographic metric originally described on lateral cervical spine radiographs to assess craniocervical junction (CCJ) instability. The ratio is calculated by dividing the shortest distance between the inferior cortex of the posterior arch of C1 and the superior cortex of the C2 spinous process by the shortest distance between the inferior cortex of the C2 spinous process and the superior cortex of the C3 spinous process (Fig. 5). An elevated C1–2:C2–3 ratio was significantly associated with tectorial membrane disruption. In their study of 71 pediatric patients, Sun et al. [24] reported that a threshold ratio ≥2.5 demonstrated 86% sensitivity and 100% specificity for MRI-confirmed tectorial membrane injury (Fig. 6). Although initially described on plain radiographs, this measurement can be reliably extrapolated to CT, where sagittal reformats provide superior visualization of bony cortices and enable precise assessment of interspinous distances.

### 3. Anterior atlanto-dental interval

**Fig. 7:**
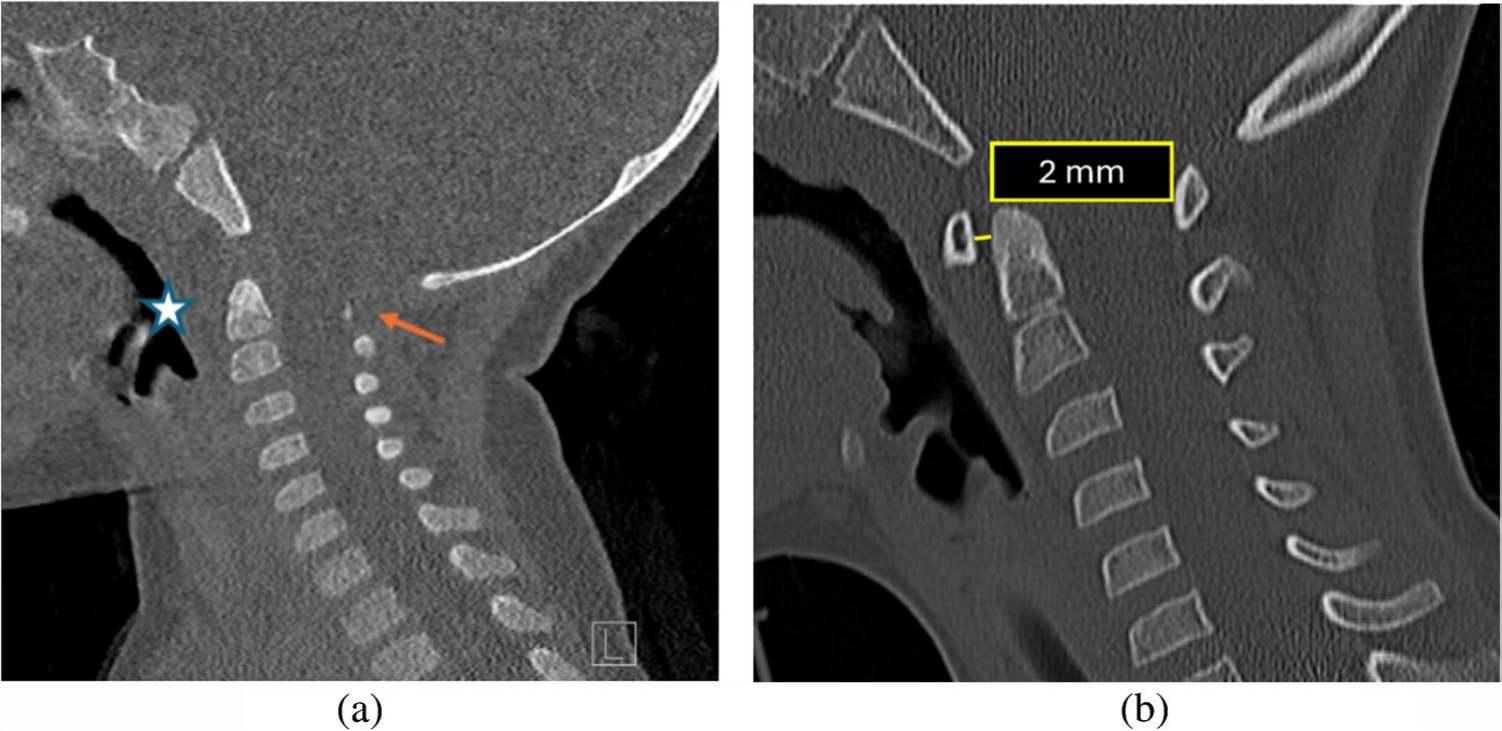
Sagittal CT in a 10-month-old with suspected non-accidental trauma. (**a**) The anterior arch of the atlas is not yet ossified (*star*). Minimally ossified posterior arch of C1 is seen (*orange arrows*). In these cases,ADI cannot be measured accurately, and clinical suspicion along with secondary signs of injury on soft-tissue windows may be useful. (**b)** Sagittal CT in a 7-year-old post-trauma shows a normal ADI (normal <2.6 mm)

The atlanto-dental interval (ADI)-more specifically the anterior atlanto-dental interval-is measured on sagittal radiographs or on CT as the horizontal distance between the mid posterior cortex of the anterior arch of C1 to the mid anterior cortex of the dens of C2 (Fig. 7).

On multi-detector CT (MDCT), the upper limit of normal for the ADI in children is 2.6 mm [25]; values above this threshold may suggest ligamentous injury or instability. Measurements should be obtained in the neutral position, as flexion or extension can alter the interval. In practice, ADI measurement on CT can be challenging due to variability in ossification of the anterior arch of C1. Piatt et al. [26] reported ossification as early as 1 month of age, with some infants lacking ossification up to 22 months, highlighting developmental variability. Hence ADI solely is not an accurate measure of atlanto-axial injury especially in children <2 years of age. If it is grossly widened in the setting of trauma along with other deranged parameters, then it indicates transverse ligament injury.

In children with Down syndrome, a radiographic ADI >6 mm in the neutral position is highly suggestive of atlanto-axial instability [27].

**Fig. 8:**
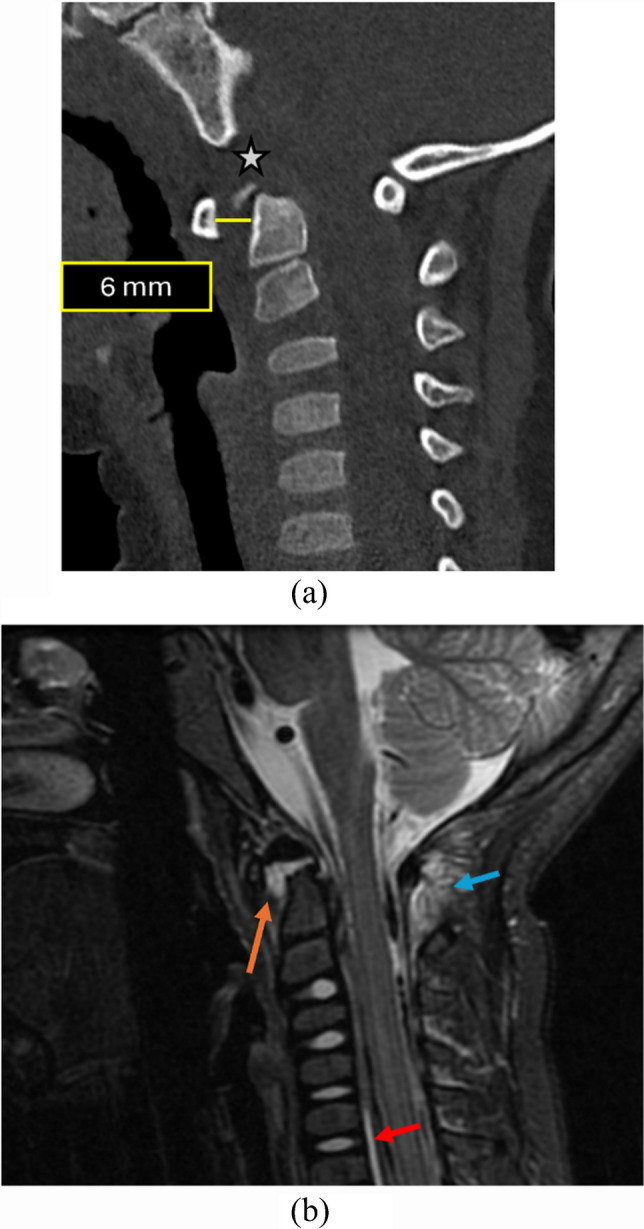
A 3-year-old child in a motor vehicle accident. (**a**) Sagittal CT of the cervical spine revealed fracture of dens (*star*) with widened anterior atlanto-dental interval measuring 6 mm (normal <2.6 mm). (**b)** MRI cervical spine performed soon after showing fluid in the anterior atlanto-dental interval (*orange arrow*). Also, ligamentous injury evidenced by edema is seen in the interspinous spaces at C1-2 (*blue arrow*). There is fluid also seen extending along the posterior longitudinal ligament (*red arrow*)

A representative case of abnormal ADI is shown in Fig. 8.

### Basion–dens interval

**Fig. 9:**
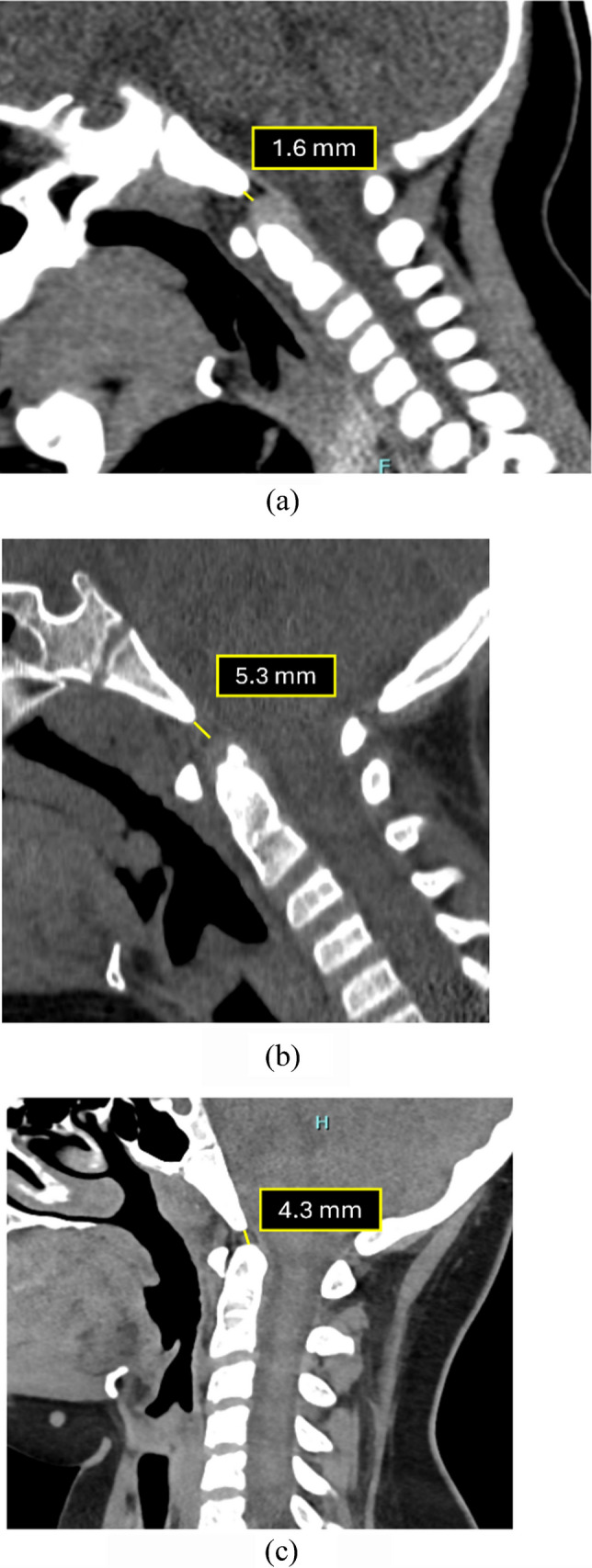
Normal measurements of BCDI and BDI in different pediatric age groups. (**a)** Sagittal CT image of a 6-month-old child. The *yellow line *indicates the normal basion–cartilaginous dens interval (BCDI), measured from the inferior-most point of the basion to the superior-most point of the cartilaginous dens, showing a value of 1.6 mm - (*N*<5.6 mm). (**b)** Sagittal CT of a 6-year-old patient following neck trauma. The *yellow line *reveals a normal BCDI of 5.3 mm (*N*<7.2 mm). At this age, the secondary ossification center of the dens is present but variable, rendering BCDI the preferred and more reliable measurement. (**c)** Sagittal CT of an 11-year-old child with near-complete ossification of the dens. The *yellow line* marks the basion–dens interval (BDI), measured between well-visualized bony landmarks, showing a value of 4.3 mm (*N*<11.6 mm)

The basion–dens interval (BDI) is the shortest midsagittal distance between the inferior margin of the basion (anterior margin of the foramen magnum) and the superior ossified tip of the dens (Fig. 9). In pediatric patients, BDI measurements are highly variable due to incomplete ossification of the dens and os terminale, resulting in inconsistent landmark identification and increased inter-observer variability [2]. Reported upper limits range from 7.49 mm to 11.6 mm depending on age and os terminale status [25, 28]. To address this limitation, Singh et al. [29] introduced the basion–cartilaginous dens interval (BCDI), which measures the cartilaginous apex when unossified. As dens ossification is largely complete by 10–12 years, the BDI becomes more reliable in older children.

However, both BDI and BCDI remain less sensitive for subtle distraction than joint-based metrics such as the condyle–C1 interval (CCI). The BDI is limited by the variable ossification of the pediatric dens (specifically the os terminale) and the fact that it spans two motion segments (C0-C1 and C1-C2), which reduces its specificity for isolated atlanto-occipital injury compared to direct joint measurements like the occipital condyle-C1 interval [23]. Furthermore, retroclival hemorrhage or epidural hematoma—often the earliest signs of injury—can obscure the cartilaginous dens tip, making this measurement difficult to perform accurately in the very cases where it is most needed.

**Fig. 10:**
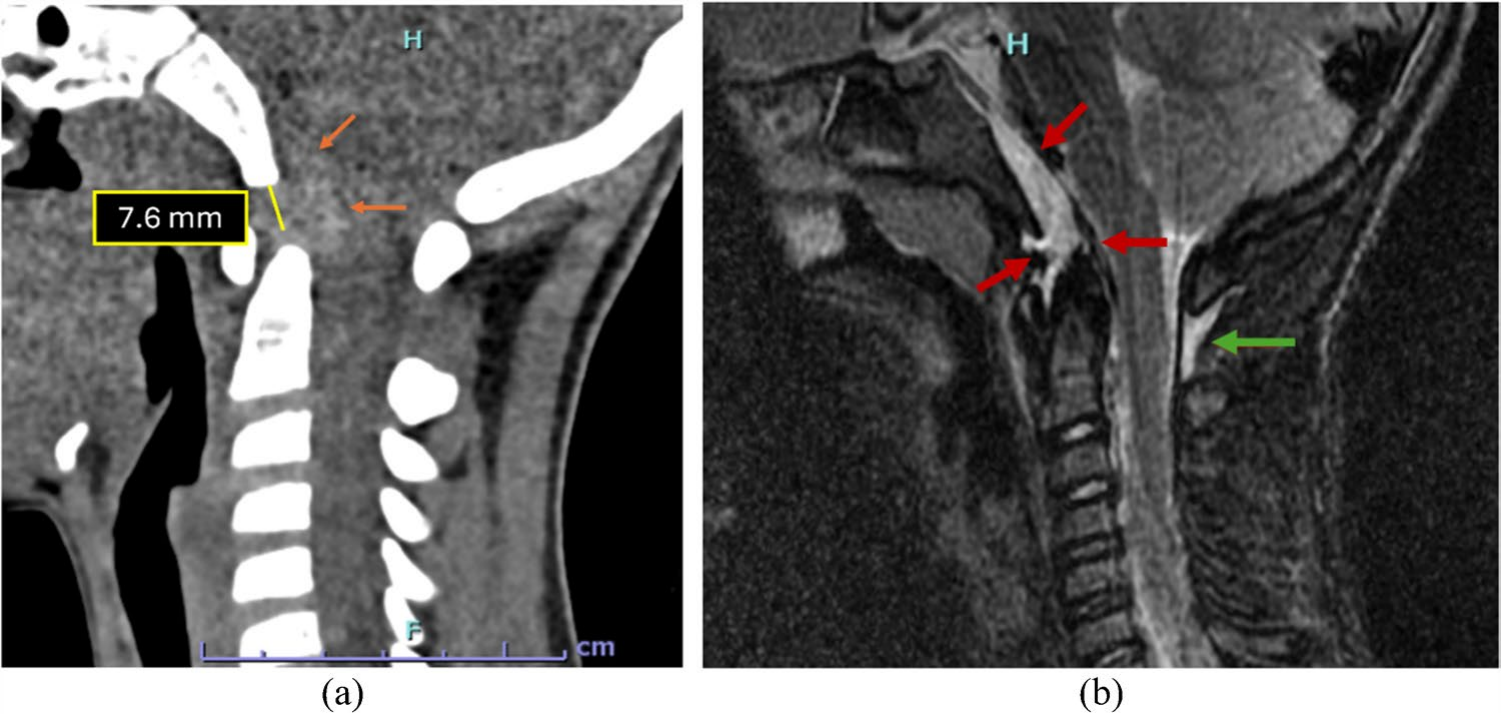
An 8-year-old child following a motor vehicle collision, presenting with neurological deterioration, neck pain, and stiffness. (**a)** Sagittal CT demonstrates an increased basion–dens interval. Given the patient’s age (<10 years), the basion–cartilaginous dens interval (BCDI) cut-off was applied (*N*<7.2 mm) with a retroclival hyperdensity (*orange arrows*) noted, raising suspicion for a distraction-type injury. (**b)** Subsequent MRI shows fluid within the basion–dens interval and stripping of the tectorial membrane from the clivus with associated hemorrhage (*red arrows*). There is also fluid seen in the C1-2 interspinous space (*green arrow*). The patient was managed with immobilization and exhibited gradual clinical recovery

Figure 10 illustrates a case from our institution in which the basion–cartilaginous dens interval was increased. Although the measurement was abnormal, several additional parameters were also deranged, collectively raising a strong suspicion for a distraction-type injury.

Table 1 summarizes the currently established age-specific upper limits for the basion–dens and basion–cartilaginous dens intervals; however, these thresholds should be utilized as supplementary screening tools, given their lower sensitivity for distraction injury.

**Table 1:** Tabular review of the cut-off values for basion-dens and basion-cartilaginous dens interval

Age group	Measurement	Upper limit
<6 years	BCDI	5.6 mm
6–10 years	BCDI	7.2 mm
10–12 years	BDI	Os terminale absent: 11.6 mm
		Os terminale present: 9.5 mm

### 5. Powers ratio

**Fig. 11:**
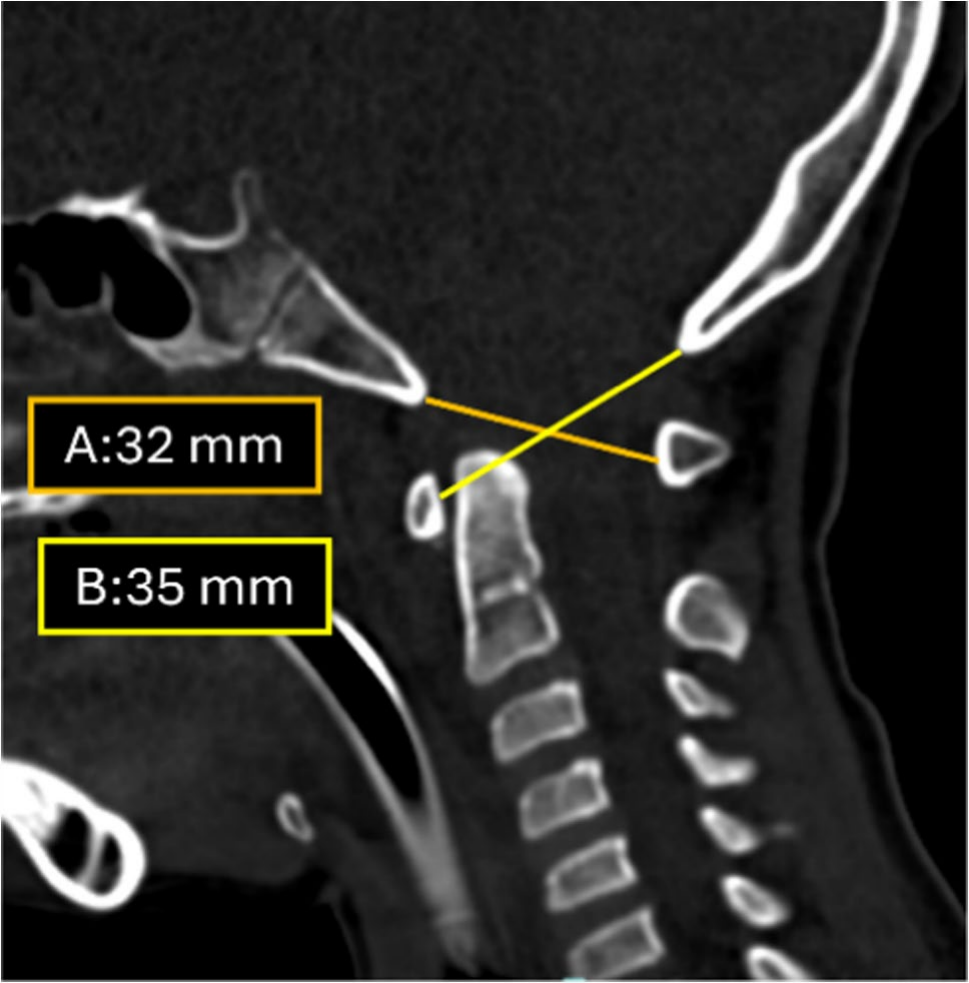
Sagittal CT in a 6-year-old with cervical trauma. Distance *A* is measured from the inferior tip of the basion to the anterior mid cortex of the posterior arch of C1 and distance *B *is measured from the inferior tip of the opisthion to the posterior mid cortex of the anterior arch of C1. Ratio of distances *A* and *B* gives the Power ratio [32/35] which is <1 (normal)

The Powers ratio is defined as the quotient of the distance from the basion to the midpoint of the posterior arch of the atlas (C1) divided by the distance from the opisthion (the posterior margin of foramen magnum) to the midpoint of the anterior arch of C1 (Fig. 11). While a ratio <1 is traditionally normal on radiographs, Bertozzi et al. [25] recommend a stricter cut-off of <0.9 for pediatric CT imaging to improve sensitivity.

The applicability of the Powers ratio can be limited in very young children with incomplete ossification of the anterior arch of C1. This ratio is also primarily sensitive to anterior atlanto-occipital dissociation. Posterior dissociation and vertical distraction injuries may yield normal ratio values, potentially leading to underdiagnosis in such cases [25]. Due to this lack of sensitivity for non-anterior injury patterns, the Powers ratio has limited diagnostic utility in modern pediatric trauma screening and should not be used in isolation to rule out craniocervical injury.

The basion-dens interval and Powers ratio serve as ancillary measurements that, while less sensitive than joint-specific metrics, often provide a critical initial clue to underlying craniocervical injury when their values deviate significantly from normative ranges. However, these traditional parameters were primarily designed to detect horizontal or anterior displacement and have been shown to be unreliable in the setting of pure vertical distraction [14]. In vertical injury patterns, the anatomical landmarks used for these ratios may maintain a deceptive spatial relationship that falls within normal limits. Therefore, radiologists should be aware that while an abnormal measurement is highly indicative of injury, a normal value does not reliably exclude the presence of craniocervical instability.

## Measurements on the coronal and axial planes

### 1. Occipital condyle–C1 interval on coronal plane

**Fig. 12:**
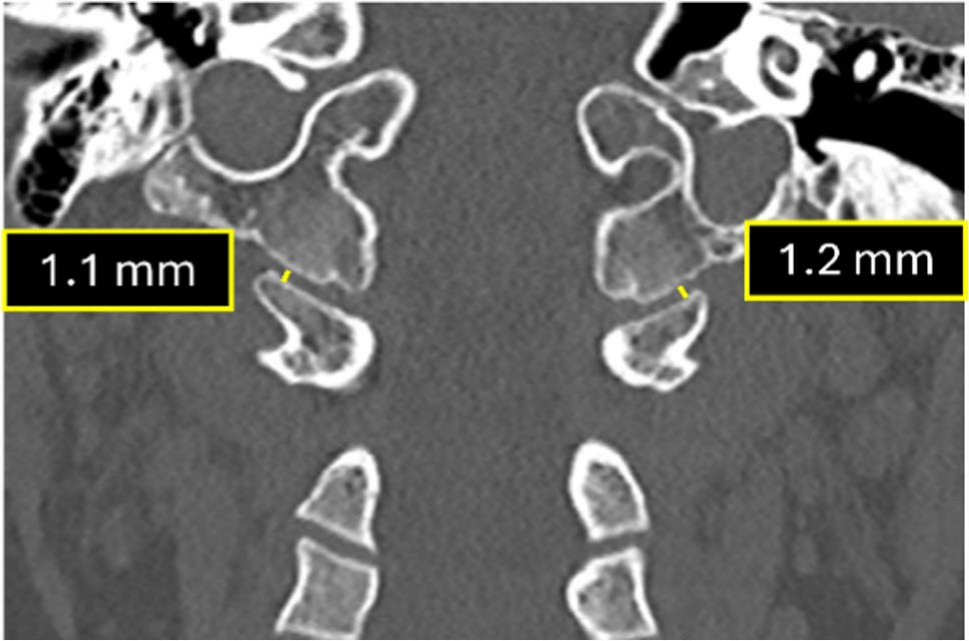
Coronal CT demonstrating normal occipital condyle–C1 interval measurements. The minimum joint space is measured between the occipital condyle and the C1 lateral mass along the lateral aspect of the joint, with exclusion of the medial occipital notch to avoid false-positive interpretation (normal <3.5 mm)

As detailed above, the occipital condyle–C1 interval (CCI) measured on coronal CT images is the valuable parameter for evaluating atlanto-occipital dissociation (AOD) (Table). The minimum joint distance should be measured at the lateral-most aspect of the articulation. To avoid false positives from the medial occipital notch, cross-referencing with sagittal images is essential to confirm the condylar surface before measuring (Fig. 12). In the large study by Ravindra et al. [21], coronal CCI ≥3.5 mm predicted AOD with 95% sensitivity, 73% specificity, 10.3% positive predictive value, and 99% negative predictive value.

**Table 2:** Summary of important measurements at the craniocervical junction with measurement technique and cut-offs

	**Measurement**	**Plane of measurement**	**How to measure**	**Upper limit**
1	Occipital condyle-C1 interval	Sagittal plane Coronal plane	*Sagittal plane*: Minimum joint space between the occipital condyle and C1 at the lateral-most aspect of the joint in the parasagittal section. Look for V-shaped widening/asymmetry of the joint space *Coronal plane*: Minimum distance in the lateral aspect of the joint space is to be measured to avoid the medial occipital notch	≥2.5 mm ≥3.5 mm
2	Atlanto-axial interval	Coronal plane	Shortest distance between the lateral mass of C1 and C2 in the lateral aspect of the joint space	2 months to 10 years: 3.9 mm 10 years to adults: 3.4 mm
3	Interspinous distance ratio	Sagittal plane	Shortest distance between the inferior cortex of the posterior arch of C1 and the superior cortex of the C2 spinous process Divided by The shortest distance between the inferior cortex of the C2 spinous process and the superior cortex of the C3 spinous process.	2.5
4	Anterior atlanto-dental interval (ADI)	Sagittal plane	Horizontal distance between the mid posterior cortex of the anterior arch of C1 to the mid anterior cortex of the dens of C2 in neutral position	≥2.6 mm
5	(a) Basion-cartilaginous dens interval (BCDI)* (	Sagittal plane	Shortest midsagittal distance between the inferior margin of the basion (anterior margin of the foramen magnum) and the superior unossified tip of the dens	<6 years: 5.6 mm 6–10 years: 7.2 mm
	(b) Basion-dens interval (BDI)*	Sagittal plane	Shortest midsagittal distance between the inferior margin of the basion and the superior tip of the dens	10–12 years If os terminale present: 9.5 mm If os terminale absent: 11.6 mm
6	Powers ratio*	Sagittal plane	Distance from the basion to the midpoint of the posterior arch of the atlas (C1) Divided by Distance from the opisthion (the posterior margin of foramen magnum) to the midpoint of the anterior arch of C1	0.9
7	Lateral atlanto-dental interval*	Coronal and axial plane	Horizontal distance between the dens and lateral mass of C1	Asymmetry can be a normal variant. Interpretation should be in conjunction with clinical findings and other impaired imaging parameters

*BCDI, BDI, Powers ratio, and lateral atlanto-dental interval have recognized limitations and should not be used in isolation to diagnose distraction injuries

**Fig. 13:**
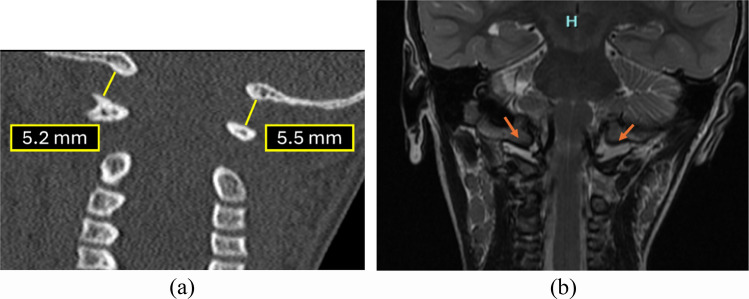
A 4-year-old child struck by a truck. (**a)** Coronal CT demonstrates an increased occipital condyle–C1 interval (normal <3.5 mm). (**b)** Corresponding coronal  T2-weighted MRI reveals fluid within the occipital condyle–C1 joint space (*orange arrows*), indicating ligamentous injury

A representative example of an abnormally increased occipital condyle-C1 interval in the coronal plane with corresponding MRI is shown in Fig. 13.

Disruption of the occipital condyle-C1 interval indicates possible injury to the capsular ligaments, which are the most important stabilizers of the craniocervical junction. The radiologist must have a high degree of suspicion in cases of high-velocity impact trauma as these injuries may prove fatal without immobilization and treatment.

### 2. Lateral atlanto-dental interval on axial and coronal

The lateral atlanto-dental interval (LADI), measured between the dens and C1 lateral masses, frequently demonstrates asymmetry in pediatric patients. Eran et al. [30] established that such asymmetry is often physiologic, typically resulting from head rotation rather than trauma. In the absence of cervical tenderness or other CT abnormalities (such as widened ADI), isolated LADI asymmetry is rarely clinically significant and generally does not warrant further imaging.

**Fig. 14:**
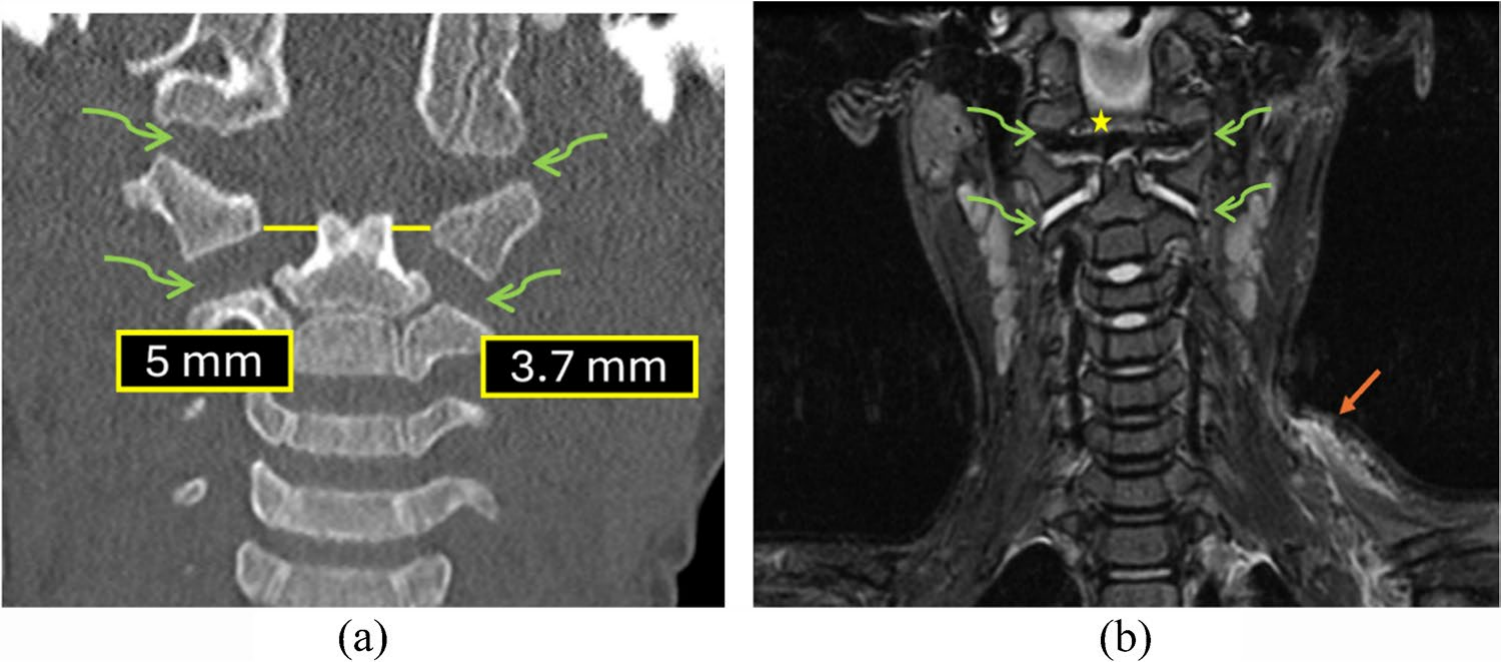
A 3-year-old child involved in a multi-vehicular accident presents with multiple limb fractures. (**a)** Coronal CT demonstrates asymmetry in the lateral atlanto-dental interval (*yellow measurements*). Given the clinical history, associated fractures, and additional evidence of craniocervical distraction injury–widening of the occipital condyle-C1 interval and atlanto-axial interval (*curved green arrows*), this finding was considered clinically significant. (**b)** Subsequent coronal MRI revealed fluid in the occipital condyle-C1 interval, and the atlanto-axial interval (*curved green arrows*). There was fluid in the visualized basion dens interval on the coronal images (star). Fluid was identified within the lateral atlantodental intervals bilaterally, corroborating the findings observed on CT. There were scattered areas of subcutaneous edema in the lower neck (*orange arrow*) indicative of soft tissue injury. The child was immobilized in a halo immediately after imaging and showed gradual clinical improvement

Currently, no standardized threshold values for LADI exist for clinically significant distraction injuries. Interpretation should be made in conjunction with clinical findings and complementary imaging parameters to ensure diagnostic accuracy (Fig. 14).

### 3. Atlanto-axial interval on coronal plane

The atlanto-axial interval (AAI), previously referred to as the lateral mass index, is defined as the shortest perpendicular distance between the lateral masses of C1 and C2. This distance is usually measured at the lateral aspect of the joint space. Historically, Gonzalez et al. [31] suggested a strict cut-off of ≥2.6 mm based on adult data. However, applying this threshold to children frequently results in false-positive diagnoses.

**Fig. 15:**
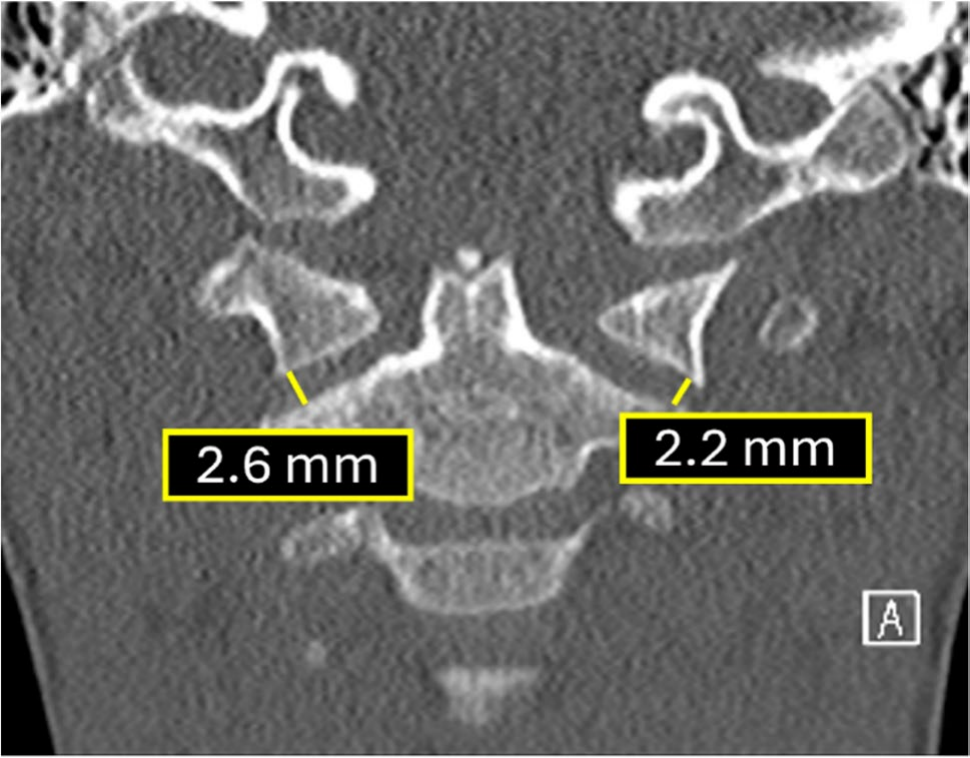
Coronal CT in a 4-year-old child demonstrates normal atlanto-axial interval

**Fig. 16:**
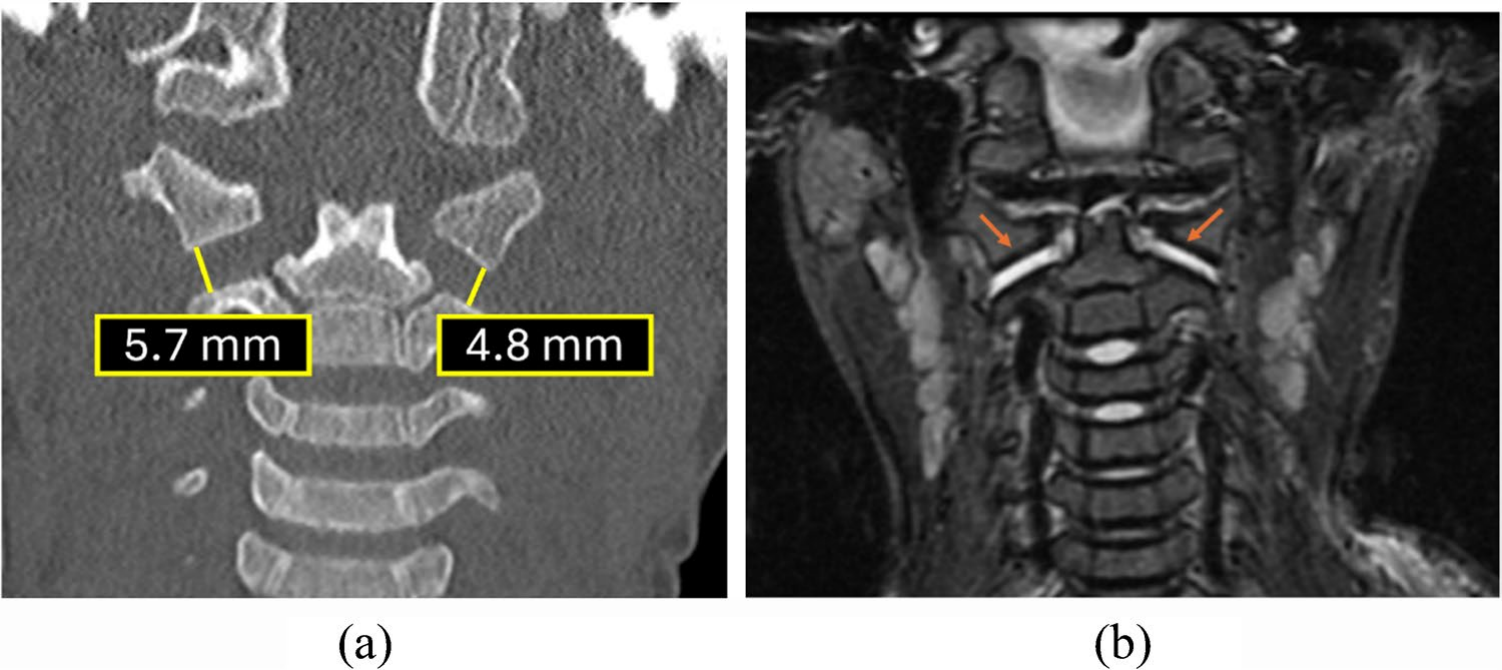
A 3-year-old child involved in a car accident. (**a)** Coronal CT demonstrates an increased atlanto-axial interval on both sides (normal <3.9 mm). The occipital condyle–C1 interval was also widened (not measured on this image). (**b)** Subsequent coronal MRI reveals edema within the occipital condyle–C1 joints and in the atlanto-axial interval (*orange arrows*), indicative of ligamentous injury

We recommend utilizing the age-specific thresholds established by Rojas et al. [32], who evaluated the C1-C2 articulation in 112 children and 178 adults. Their findings dictate the following interpretation:

*  Children (2 months–10 years)*: Due to incomplete ossification of the lateral masses, the apparent joint space is wider. The upper limit of normal (95th percentile) in this group is 3.9 mm. Values below this threshold should be considered physiologic (note: while infants <2 months were not explicitly studied, the principle of unossified cartilage suggests a similarly wide interval). Figure 15 shows the normal atlanto axial interval and it's measurement technique. Figure 16 shows a case of abnormal Atlantoaxial interval.  

  *Adolescents and adults (>10 years)*: As ossification progresses and the skeleton matures, the joint interval narrows. Rojas et al. found the upper limit in adults to be approximately 3.4 mm. Therefore, in children older than 10 years, a cut-off closer to the adult standard (3.4 mm) is appropriate, and values approaching the pediatric 3.9 mm limit should be viewed with higher suspicion.

##  The key craniocervical junction measurements including their measurement planes, techniques and accepted upper limits across age groups is summarised in table 2 (Table 2)Case examples

The utility of these measurements is exemplified by two illustrative cases from our institution, shown with corresponding CT and MRI images.

**Fig. 17:**
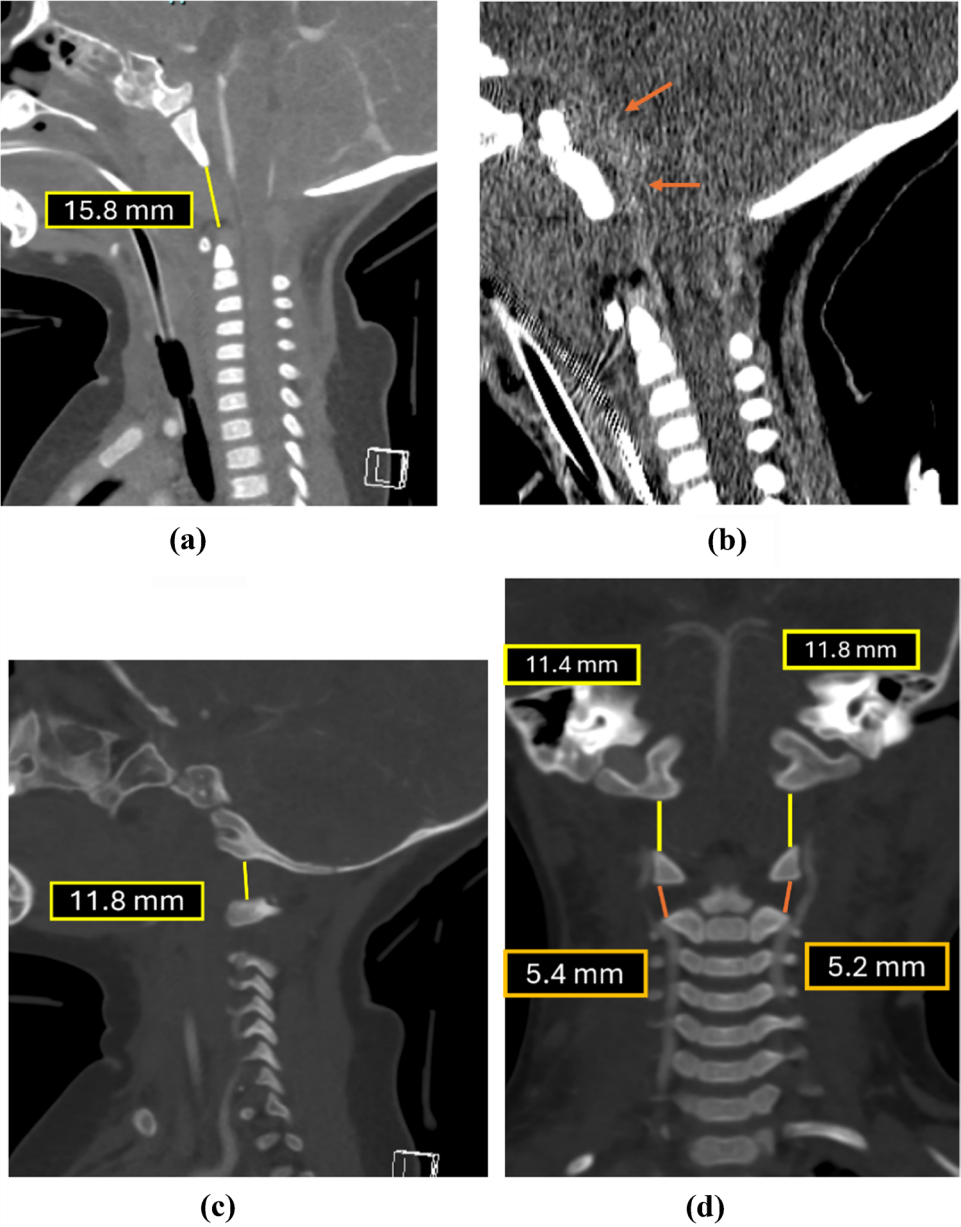
The initial CT was interpreted as normal, with no evidence of fracture. On detailed review, the following findings were noted: (**a**) basion–cartilaginous dens interval (BCDI) was markedly increased at * 15.8  mm* (normal upper limit for age <6 years: *5.6 mm*); (**b**) retroclival hyperdensity became evident after adjusting window settings (*orange arrows*); (**c**) parasagittal CT images demonstrated a significantly widened  occipital condyle -C1 interval with V-shaped widening of the joint space and elevated distance (normal <2.5 mm); (**d**) coronal CT images revealed both an increased occipital–condyle-C1 interval (normal <3.5 mm) and elevated atlanto-axial interval (normal <3.9 mm). No fractures were identified

**Fig. 18:**
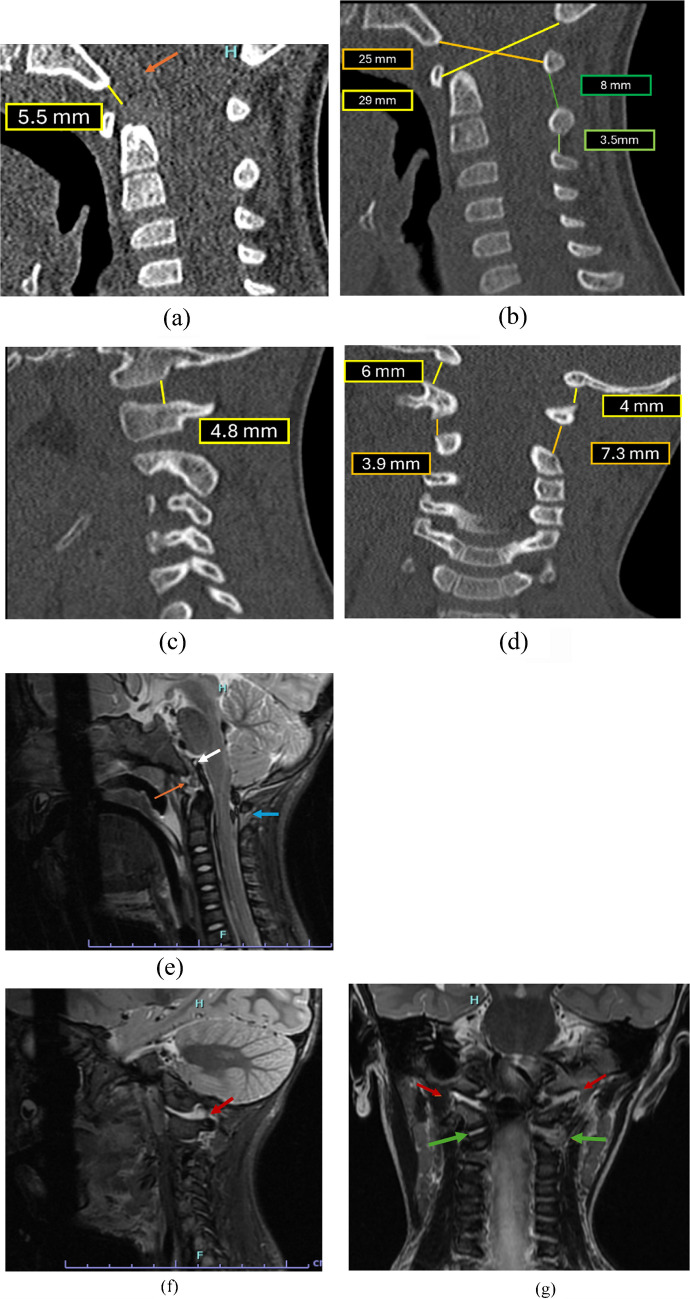
**(a**, **b)** Sagittal CT images demonstrate normal BBasion Cartilaginous Dens Interval  (BCDI) measuring 5.5 mm [N<5.6 mm] and Powers ratio which measured 0.8 [N<0.9]. A subtle retroclival hyperdensity is noted (*orange arrows*), though characterization is limited on CT. The interspinous distance ratio measures 2.3 which is also within normal limits [N<2.5]  (**c**, **d)** Sagittal and coronal CT images reveal asymmetry of the occipital condyle–C1 interval as shown in the right parasagittal region measuring 4.8 mm  and on the coronal images measuring 6 mm on the right and 4 mm on the left (*N*<2.5 mm on sagittal and <3.5 mm on coronal). The atlantoaxial interval is also increased on both sides measuring 3.9 mm on the right and 7.3 mm on the left (N<3.9 mm). Subsequent MRI findings: (**e**) sagittal T2 fat saturated images of the cervical spine  demonstrates elevation of the tectorial membrane (*white arrow*) with thin interposed fluid layer between the membrane and clivus, without complete detachment. There is edema in the basion-dens interval (*orange arrow*) and in the interspinous region between C1 and C2 (*blue arrow*) without significant widening indicative of soft tissue edema. (**f**) Parasagittal T2 fat saturated images revealed T2 hyperintensity within the occipital condyle–C1 (*red arrows*) with V shaped widening. (**g)** Coronal T2 fat saturated images confirm widening of the occipital condyle–C1 interval on both sides with fluid in these regions (*red arrows*). There is widening with fluid also seen in bilateral atlantoaxial intervals (*green arrows*), consistent with craniocervical distraction injury

## Case 1

An 8-month-old infant was involved in a motor vehicle collision. Initial CT imaging of the head and cervical spine was performed at an outside hospital as part of the trauma evaluation. The patient was subsequently referred to our institution for further management. On arrival, the neurological status was markedly impaired (Fig 17)

Overall, these findings are consistent with atlanto-occipital distraction injury, characterized by tectorial membrane disruption (suggested by retroclival hemorrhage), increased BDCI, widened occipital condyle-C1 interval, and atlanto-axial interval indicating disruption of capsular ligaments. Minimal evidence of anterior atlanto-axial dislocation was present. The patient unfortunately succumbed to injuries shortly after imaging.

## Case 2

A 27-month-old child presented to the emergency department in a lethargic state following a motor vehicle collision, with fluctuating levels of consciousness and waxing and waning Glasgow Coma Scale (GCS). Initial cervical spine CT demonstrated subtle retroclival hyperdensity without evidence of fracture or malalignment. Given persistent neurological asymmetry and high clinical concern for craniocervical distraction injury, the imaging was re-evaluated with focused attention to craniocervical junction parameters (Fig 18).

Following these findings, the patient was immobilized in a halo device and subsequently demonstrated progressive clinical improvement. This case further illustrates that the Occipital condyle-C1 interval and Atlantoaxial intervals represent more sensitive imaging parameters for the identification of clinically significant distraction injuries.

## Conclusion

Based on our institutional experience and retrospective case analysis, we emphasize the importance of a structured and anatomy-driven approach to the evaluation of craniocervical junction (CCJ) trauma. In high-impact mechanisms or when neurological findings appear disproportionate to initial imaging, the systematic application of standardized CCJ measurements on CT is essential, even in the absence of overt fractures, to avoid missed ligamentous injuries.

The ligaments most critical to craniocervical stability—and therefore most relevant in the assessment of distraction-type injuries—include the capsular ligaments of the atlanto-occipital and atlanto-axial joints, the alar ligaments, the transverse ligament, and the tectorial membrane. Accordingly, diagnostic emphasis should shift away from less sensitive traditional metrics such as the Powers ratio and basion–dens interval toward coronal plane assessment of the occipital condyle–C1 interval (CCI) and atlanto-axial interval (AAI), which more directly interrogate the integrity of these primary stabilizers.

Ancillary imaging findings, including retroclival or periligamentous hyperdensities on axial CT, can provide supportive evidence of ligamentous injury and should prompt heightened suspicion, particularly in subtle dissociation injuries. While secondary parameters may not independently define instability, their abnormalities can contribute valuable contextual information when interpreted alongside targeted CCJ measurements.

Heightened awareness, consistent application of these imaging principles, and prioritization of ligament-specific metrics can significantly improve diagnostic accuracy and facilitate timely management, ultimately contributing to better clinical outcomes in pediatric patients with suspected CCJ injuries.

### Role of dynamic imaging

In children with normal static CT findings who present with persistent neck pain (subacute setting), flexion-extension radiographs serve as a valuable problem-solving tool to exclude occult ligamentous instability [33]. Current consensus guidelines emphasize that this dynamic assessment is reserved for alert, cooperative patients capable of active, voluntary motion. The study is only considered diagnostic if the patient achieves adequate excursion (≥30° of total flexion-extension). Adequacy is confirmed quantitatively via C2–C7 Cobb angles or qualitatively by observing fanning (widening) of the spinous processes during flexion and their approximation during extension.

When discrepancies in measurements or suspicious findings are identified, MRI should be recommended to confirm and further characterize ligamentous integrity. The radiologist’s vigilance in recognizing subtle imaging abnormalities is essential for timely diagnosis and prevention of potentially severe neurological sequelae.

### Recommendations and future direction

The craniocervical junction measurements presented in this review were compiled from established literature and prior studies to provide a consolidated reference for radiologists in clinical practice. While these parameters offer valuable guidance, further research involving pediatric trauma populations with pathological correlation is necessary to validate the proposed thresholds, particularly across all pediatric ages, and support their routine diagnostic application.

## Data Availability

No datasets were generated or analysed during the current study.
